# Robot Assisted Training for the Upper Limb after Stroke (RATULS): study protocol for a randomised controlled trial

**DOI:** 10.1186/s13063-017-2083-4

**Published:** 2017-07-20

**Authors:** Helen Rodgers, Lisa Shaw, Helen Bosomworth, Lydia Aird, Natasha Alvarado, Sreeman Andole, David L. Cohen, Jesse Dawson, Janet Eyre, Tracy Finch, Gary A. Ford, Jennifer Hislop, Steven Hogg, Denise Howel, Niall Hughes, Hermano Igo Krebs, Christopher Price, Lynn Rochester, Elaine Stamp, Laura Ternent, Duncan Turner, Luke Vale, Elizabeth Warburton, Frederike van Wijck, Scott Wilkes

**Affiliations:** 1 Stroke Research Group, Institute of Neuroscience, Newcastle University, 3-4 Claremont Terrace, Newcastle upon Tyne, NE2 4AE UK; 2 Stroke Northumbria, Northumbria Healthcare NHS Foundation Trust, North Tyneside General Hospital, Rake Lane, North Shields, Tyne and Wear NE29 8NH UK; 3 Institute of Health and Society, Newcastle University, Baddiley-Clark Building, Richardson Road, Newcastle upon Tyne, NE2 4AX UK; 4 Barking, Havering and Redbridge University Hospitals NHS Trust Queen’s Hospital, Rom Valley Way, Romford, Essex RM7 0AG UK; 5 North West London Hospitals NHS Trust, Northwick Park Hospital, Watford Road, Harrow, HA1 3UJ UK; 6 University of Glasgow, Queen Elizabeth University Hospital, 1342 Govan Road, Govan, Glasgow, G51 4TF UK; 7 Department of Child Health, Institute of Neuroscience, Newcastle University, Royal Victoria Infirmary, Queen Victoria Road, Newcastle upon Tyne, NE1 4LP UK; 8 Medical Sciences Division, University of Oxford, and Oxford University Hospitals NHS Foundation Trust, Oxford, OX3 9DU UK; 9 Oxford Academic Health Science Network, Magdalen Centre North Oxford Science Business Park, Oxford, OX4 4GA UK; 10 Health Economics Group, Institute of Health and Society, Newcastle University, Baddiley-Clark Building, Richardson Road, Newcastle upon Tyne, NE2 4AX UK; 11 Contact via: Stroke Research Group, Institute of Neuroscience, Newcastle University, 3-4 Claremont Terrace, Newcastle upon Tyne, NE2 4AE UK; 12 NHS Greater Glasgow and Clyde, Queen Elizabeth University Hospital, 1342 Govan Road, Govan, Glasgow, G51 4TF UK; 13 Massachusetts Institute of Technology, 77 Massachusetts Avenue, 3-137, Cambridge, MA 02139 USA; 14 Stroke Northumbria, Northumbria Healthcare NHS Foundation Trust, Wansbeck General Hospital, Woodhorn Lane, Ashington, Northumberland NE63 9JJ UK; 15 Institute of Neuroscience, Newcastle University, Clinical Ageing Research Unit, Campus for Ageing and Vitality, Newcastle upon Tyne, NE4 5PL UK; 16 University of East London, School of Health, Sport and Biosciences, Stratford Campus, Water Lane, Stratford, London, E15 4LZ UK; 17 Cambridge University Health Partners (Addenbrooke’s Hospital), R3 Neurosciences, Addenbrooke’s Hospital, Hills Road, Box 83, Cambridge, CB2 2QQ UK; 18 Institute for Applied Health Research and School of Health and Life Sciences, Glasgow Caledonian University, Cowcaddens Road, Glasgow, G4 0BA UK; 19 Department of Pharmacy, Health and Wellbeing, Faculty of Applied Sciences, Science Complex, University of Sunderland, City Campus, Chester Road, Sunderland, SR1 3SD UK

**Keywords:** Stroke, Arm, Rehabilitation, Robotics, RCT, Cost-effectiveness analysis, Parallel process evaluation

## Abstract

**Background:**

Loss of arm function is a common and distressing consequence of stroke. We describe the protocol for a pragmatic, multicentre randomised controlled trial to determine whether robot-assisted training improves upper limb function following stroke.

**Methods/design:**

Study design: a pragmatic, three-arm, multicentre randomised controlled trial, economic analysis and process evaluation.

Setting*:* NHS stroke services.

Participants: adults with acute or chronic first-ever stroke (1 week to 5 years post stroke) causing moderate to severe upper limb functional limitation.

Randomisation groups:

1. Robot-assisted training using the InMotion robotic gym system for 45 min, three times/week for 12 weeks

2. Enhanced upper limb therapy for 45 min, three times/week for 12 weeks

3. Usual NHS care in accordance with local clinical practice

Randomisation: individual participant randomisation stratified by centre, time since stroke, and severity of upper limb impairment.

Primary outcome: upper limb function measured by the Action Research Arm Test (ARAT) at 3 months post randomisation.

Secondary outcomes: upper limb impairment (Fugl-Meyer Test), activities of daily living (Barthel ADL Index), quality of life (Stroke Impact Scale, EQ-5D-5L), resource use, cost per quality-adjusted life year and adverse events, at 3 and 6 months.

Blinding: outcomes are undertaken by blinded assessors.

Economic analysis: micro-costing and economic evaluation of interventions compared to usual NHS care. A within-trial analysis, with an economic model will be used to extrapolate longer-term costs and outcomes.

Process evaluation: semi-structured interviews with participants and professionals to seek their views and experiences of the rehabilitation that they have received or provided, and factors affecting the implementation of the trial.

Sample size: allowing for 10% attrition, 720 participants provide 80% power to detect a 15% difference in successful outcome between each of the treatment pairs. Successful outcome definition: baseline ARAT 0–7 must improve by 3 or more points; baseline ARAT 8–13 improve by 4 or more points; baseline ARAT 14–19 improve by 5 or more points; baseline ARAT 20–39 improve by 6 or more points.

**Discussion:**

The results from this trial will determine whether robot-assisted training improves upper limb function post stroke.

**Trial registration:**

ISRCTN, identifier: ISRCTN69371850. Registered 4 October 2013.

**Electronic supplementary material:**

The online version of this article (doi:10.1186/s13063-017-2083-4) contains supplementary material, which is available to authorized users.

## Background

Stroke is the commonest cause of complex adult disability in high-income countries [1]. Loss of arm function affects 69% of people who have a stroke [2]. Only 12% of people with arm weakness at the onset of stroke make a full recovery [3]. Improving arm function has been identified as a research priority by stroke survivors, carers and health professionals who report that current rehabilitation pays insufficient attention to arm recovery [4].

Robot-assisted training enables a greater number of repetitive tasks to be practised in a consistent and controllable manner. Repetitive task training is known to drive Hebbian plasticity, where wiring of pathways that are coincidently active is strengthened [5, 6]. A dose of greater than 20 h of repetitive task training improves upper limb motor recovery following a stroke [7] and, therefore, robot-assisted training has the potential to improve arm motor recovery after stroke. We anticipate that Hebbian neuroplasticity, which is learning dependent, will operate regardless of the post-stroke phase.

A Cochrane systematic review of electromechanical and robot-assisted arm training after stroke reported outcomes from a total of 1160 patients who participated in 34 randomised controlled trials (RCTs). Improvements in arm function (standardised mean difference (SMD) 0.35, 95% confidence interval (CI), 0.18–0.51) and activities of daily living (SMD 0.37, 95% CI, 0.11–0.64) were found in patients who received this treatment, but studies were often of low quality [8]. In the UK there is currently insufficient evidence to justify the use of this technology in routine clinical practice.

In addition, studies which suggest that robot-assisted training may improve upper limb function after stroke should be treated with caution as participants who were randomised to receive robot-assisted training may have also received an increased intensity of rehabilitation sessions (e.g. frequency or duration) compared to participants in the control groups. Greater intensity of upper limb rehabilitation sessions has been shown to improve upper limb functional outcomes [7], and a meta-analysis of robot-assisted training RCTs reported that if control group therapy sessions were delivered at the same frequency and duration, there was no additional functional improvement [9]. Studies are required which provide further direct evidence of the effectiveness of robot-assisted training without the confounding effect of therapy dose.

The aim of the Robot Assisted Training for the Upper Limb after Stroke (RATULS) trial is to evaluate the clinical and cost-effectiveness of robot-assisted training compared to an upper limb therapy programme of the same frequency and duration, and usual post-stroke care.

The null hypothesis is that there is no difference in upper limb function at 3 months between study participants who receive robot-assisted training and those who receive an enhanced upper limb therapy programme and those who receive usual post-stroke care. The RATULS trial will be making comparisons of the effectiveness of rehabilitation on upper limb function between all three pairs of trial arms.

## Methods

### Study aim and objectives

#### Aim

To determine whether robot-assisted training with the InMotion robotic gym system (InMotion commercial version) improves upper limb function post stroke.

#### Objectives

- To determine whether robot-assisted training improves upper limb function post stroke compared to an enhanced upper limb therapy programme or usual care
- To determine whether robot-assisted training improves upper limb impairment, activities of daily living and quality of life compared to an enhanced upper limb therapy programme or usual care
- To model the costs of robotic-assisted training compared to an enhanced upper limb therapy programme or usual care
- To seek the views and experiences of patients and health service professionals about the upper limb rehabilitation that they have received or provided and factors affecting the implementation of the trial
- To explore:
  - the time pattern of upper limb recovery of participants in each treatment group
  - the impact of the severity of baseline upper limb function and time since stroke upon the effectiveness of the interventions

### Study design

**Fig. 1:**
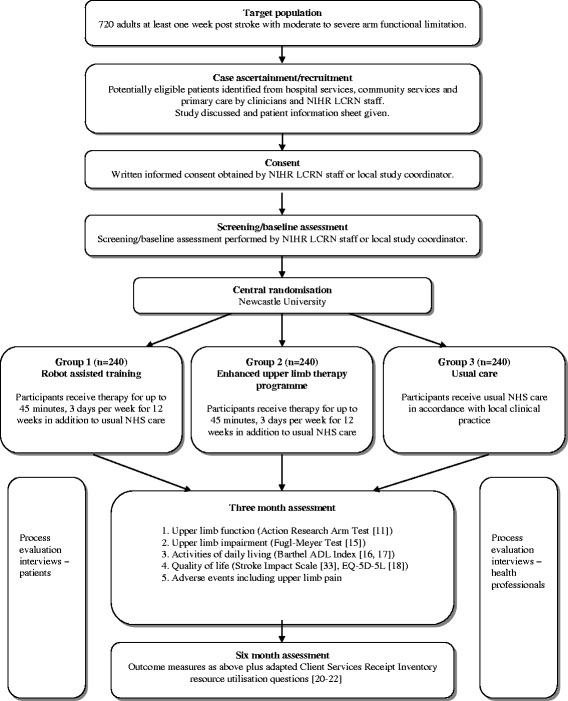
RATULS trial summary

**Fig. 2:**
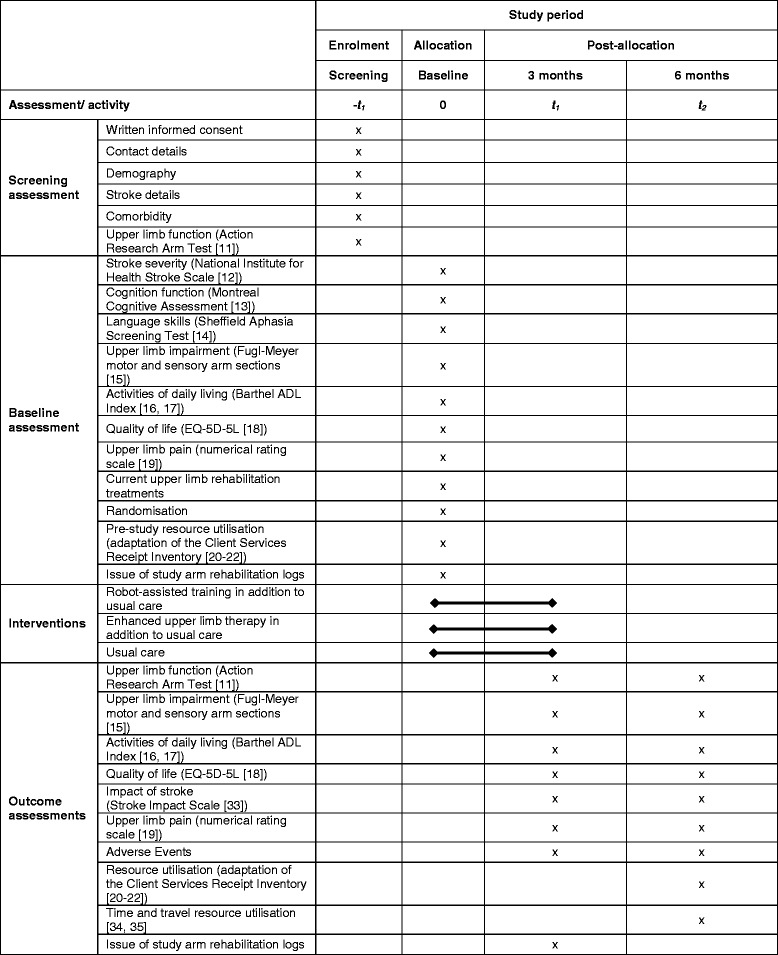
Standard Protocol Items: Recommendations for Interventional Trials (SPIRIT) schedule of enrolment, interventions and assessments

This study is a three-arm, pragmatic, observer-blind, multicentre RCT with embedded economic analysis and a process evaluation. Participants are randomised to receive either: robot-assisted training (in addition to usual NHS care); an enhanced upper limb therapy programme (in addition to usual NHS care); or usual NHS care in accordance with local clinical practice. Figure 1 summarises the study methods. The study is presented according to the Standard Protocol Items: Recommendations for Interventional Trials (SPIRIT) [10] (SPIRIT Checklist, Additional file 1). Figure 2 shows the SPIRIT schedule of enrolment, interventions and assessments.

### Study setting

The study is being conducted in NHS stroke units in the UK. There are four RATULS study centres (Glasgow, North Tyneside, Northwick Park, and Romford) each consisting of a hub site with an InMotion robotic gym system and spoke sites which are stroke services in adjacent trusts that refer patients to take part in the study and provide usual NHS care.

### Study participants

Adults with a first-ever stroke who fulfil the following criteria are eligible to participate in the trial:

#### Inclusion criteria

- Age 18 years and over
- Clinical diagnosis of stroke (cerebral infarction, primary intracerebral haemorrhage, subarachnoid haemorrhage)
- Between 1 week and 5 years since stroke
- Moderate to severe upper limb functional limitation (Action Research Arm Test (ARAT) [11] score 0–39) due to stroke
- Able to provide consent to take part in the study and to comply with the requirements of the protocol

#### Exclusion criteria

- More than one stroke (patients with previous transient ischaemic attack (TIA) may be invited to participate)
- Other current significant impairment of the upper limb affected by stroke, e.g. fixed contracture, frozen shoulder, severe arthritis, recent fracture
- Diagnosis likely to interfere with rehabilitation or outcome assessments, e.g. registered blind
- Previous use of the InMotion robotic gym system or other arm rehabilitation robot
- Current participation in a rehabilitation trial evaluating upper limb rehabilitation after stroke
- Previous enrolment in the RATULS study

### Case ascertainment and recruitment

Study participants are recruited from both incident and prevalent stroke populations. Participants can be sought from a number of settings in both primary and secondary care including: stroke units; outpatient clinics; day hospitals; community rehabilitation services; and general practices. The study aims to recruit similar numbers of participants within: 0–3 months of stroke; >3–12 months after stroke; and >12 months to 5 years after stroke.

#### Potential participants from secondary care

In secondary care, potential participants are identified by local clinicians and/or staff from the National Institute for Health Research Local Clinical Research Network (NIHR LCRN). Staff approach potentially eligible patients, discuss the study and provide a study information leaflet. After allowing sufficient time for the information to be considered, staff ask the patient if they are potentially interested in taking part in the study.

Potential participants can also be identified from hospital stroke discharge summaries/clinic letters. If this method is used, potential participants are approached by letter. Enclosed with the letter is a short RATULS leaflet, a Patient Information Sheet, a RATULS reply slip and a pre-paid envelope. Interested patients may make contact with the study centre by telephone or by return of the RATULS reply slip. Following a few short telephone questions to confirm potential study eligibility, a face-to-face appointment for further discussion is subsequently arranged if appropriate.

#### Potential participants from primary care

To identify potential participants from primary care, general practices perform a database search using the study inclusion/exclusion criteria. A GP screens the list of potentially eligible participants to approve the issue of an invitation letter. This letter is accompanied by the same information which is sent to individuals identified from secondary care records. The invitation letter details the main study eligibility criteria and asks interested patients to contact the study centre for further information. Following a few short telephone questions to confirm potential study eligibility, a face-to-face appointment for further discussion is subsequently arranged if appropriate.

#### Potential patients from other sources

Local community stroke clubs and day centres are also given information about the study. In addition, some individuals may hear about the study from press releases or see information about the study on a poster or RATULS leaflet. Interested individuals are able to contact the study centres directly for a discussion about the study.

### Consent

Individuals who are interested and potentially eligible to take part in the study are given an appointment for further discussion and consent. This may be conducted by a local study coordinator or NIHR LCRN staff. Written informed consent is obtained if the patient wishes to take part in RATULS.

### Screening log

A screening log is kept at each study centre. This records details of all inpatients, outpatients and primary care patients considered for the study and subsequently included or excluded.

### Screening assessment

Once written informed consent is obtained, a screening assessment is performed by the local study centre coordinator or NIHR LCRN staff. The following data are collected: demography; stroke details; comorbidity; and upper limb function (ARAT score [11]). If the patient fulfils the study inclusion and exclusion criteria, the local study coordinator/NIHR LCRN staff proceeds to the baseline assessment. If it is not possible to complete the baseline assessment on the same day as the screening assessment, eligibility for the study is re-confirmed on the day of the baseline assessment.

### Baseline assessment

The following baseline data are collected: stroke severity (National Institute for Health Stroke Scale [12]); cognitive function (Montreal Cognitive Assessment [13]); language skills (Sheffield Aphasia Screening Test [14]); upper limb impairment (Fugl-Meyer Test (motor and sensory arm sections) [15]); activities of daily living (Barthel ADL Index [16, 17]; quality of life (EQ-5D-5 L [18]); upper limb pain (numerical rating scale [19]) and current upper limb rehabilitation treatments. In addition, patients are given a self-completion questionnaire containing pre-study resource utilisation questions (adaption of the Client Services Receipt Inventory [20–22]) which they are asked to complete at the end of the assessment.

### Randomisation

Randomisation is conducted by the local study coordinator/NIHR LCRN staff following completion of the baseline assessment. A central independent web-based service hosted by Newcastle University Clinical Trials Unit is used. Participants are stratified according to study centre, time since stroke and severity of upper limb function (ARAT score [11]), and randomised to either robot-assisted training, enhanced upper limb therapy, or usual care groups using permuted block sequences.

### Randomisation groups

#### Robot-assisted training using the InMotion robotic gym system

This is delivered using the InMotion robotic gym system which was specifically designed for clinical rehabilitation applications [23–25]. This is currently the most widely used technology for robot-assisted training for patients with moderate to severe upper limb impairment post stroke. The system development started in 1989 and it has amassed the largest body of clinical evidence to date of any robotic system. It has been successfully tested in clinical studies involving over 900 stroke patients and there are around 250 robots in use worldwide.

Participants receive robot-assisted training at the hub sites for up to 45 min per day, three days per week for 12 weeks, in addition to usual care. A detailed description of the robot-assisted training programme using the Template for Intervention Description and Replication (TIDieR) Checklist [26] is provided in Table 1.

**Table 1:** Template for Intervention Description and Replication (TIDieR) Checklist for the RATULS trial intervention treatments

Item	Intervention group	
1. Brief name: *‘provide the name or phrase that describes the intervention’*	*Robot-assisted training*	*Enhanced upper limb therapy*
2. Why?: *‘describe any rationale, theory, or goal of the elements essential to the intervention’*	*Rationale for robot-assisted training:* Increased intensity of rehabilitation is known to improve functional outcomes and moderate-quality evidence shows repetitive task training (when dose >20 h) increases arm function post stroke [7]. Robot-assisted training enables repetitive tasks to be undertaken in a highly consistent and controllable manner. A Cochrane systematic review of electromechanical and robot-assisted arm training after stroke reported outcomes from a total of 1160 patients who participated in 34 trials. Improvements in arm function (SMD 0.35, 95% confidence interval (CI), 0.18–0.51) and activities of daily living (SMD 0.37, 95% CI 0.11–0.64) were found in patients who received this treatment, but studies were often of low quality [8] *Essential elements:* • Robotic device • Repetitive task practice • Increased intensity of training	*Rationale for enhanced upper limb therapy:* Increased intensity of rehabilitation is known to improve functional outcomes and moderate-quality evidence shows repetitive task training (when dose >20 h) increases arm function post stroke [7]. In addition, evidence suggests that patients benefit most from exercise programmes that involve functional tasks which are directly practised [46]. Motivation and engagement in therapy can be increased by goal-setting and monitoring goal achievement [47]. *Essential elements:* • Repetitive functional task practice • Patient-centred goal-setting • Increased intensity of training
3. What materials?: *‘describe any physical or informational materials used in the intervention, including those provided to participants or used in intervention delivery or in training of intervention providers. Provide information on where the materials can be accessed’*	InMotion robotic gym system is used (http://bionikusa.com/healthcarereform/upper-extremity-rehabilitiation/). The robotic gym consists of three robot modules to train the participant to use their upper limb: • The shoulder-elbow module (InMotion ARM^TM^ interactive therapy system). The participant moves their affected arm radially in eight different directions • The wrist module (InMotion WRIST^TM^ interactive therapy system). The movements of the participant’s wrist include flexion/extension, abduction/adduction and pronation/supination • The hand module integrated onto the shoulder-elbow module **(**InMotion HAND^TM^). This encourages whole-arm movements that involve limb transport and grasp/release. Study-specific manuals describing robot-assisted training were produced and are used by staff delivering therapy. Study-specific documentation is used by therapists to record attendance at sessions. The robot software records data on the robot protocol used, duration of the sessions and the number of repetitions of upper limb movements undertaken. Staff delivering the robot-assisted training programme receive specific training	The enhanced upper limb therapy programme consists of repetitive functional task practice aimed at patient-centred goals. It has been developed from upper limb therapy programmes used in the Botulinum Toxin for the Upper Limb after Stroke (BoTULS) trial [27–29] and the Repetitive Arm Functional Tasks after Stroke (RAFTAS) project [30]. Study-specific manuals describing enhanced upper limb therapy were produced and are used by staff delivering therapy. Included in the manual is a list of potential goals and a description of suggested activities for each goal. Everyday items to enable functional task practice are provided. Study-specific documentation is used by therapists to record session attendance, session duration, the type and number of goals, the type of activity practice (‘whole-task’ or ‘part-task’) and number of repetitions of each task at each session. Goal attainment is documented at each review session. Staff delivering the enhanced upper limb therapy programme receive specific training
4. What (procedures)?: *‘describe each of the procedures, activities and/or processes used in the intervention, including any enabling support activities’*	The robot-assisted training programme is divided into three consecutive blocks in order to integrate training with all three robot modules. Training sessions on all robot modules consist of high repetitions (aiming for >700 per session) of point-to-point movements. Block 1: block one lasts for 2 weeks and employs alternate training sessions with the shoulder-elbow module and the wrist module (three sessions on each robot module). The robot modules rhythmically move the participant’s upper limb to reach sequentially presented targets. Block 2: block 2 lasts for 6 weeks and employs alternate therapy sessions with the shoulder-and-elbow and the wrist modules (9 sessions on each robot module). The robot modules allow the participant to attempt to move towards sequentially presented targets unassisted but will assist if the participant needs help to reach the target. Block 3: block 3 lasts for 4 weeks and employs alternative therapy sessions with the hand module integrated on the shoulder-elbow module, and the wrist module (6 sessions on each module). As in block 2, the robot modules allow the participant to attempt to move towards the targets unassisted but will assist if the participant needs help to reach the target. For the therapy sessions with the hand module integrated on the shoulder-elbow module, targets are presented sequentially. For the therapy sessions with the wrist module, the targets are presented randomly. Evaluations of robotic kinematics (i.e. related to the movement pattern) and kinetics (i.e. related to the causes of movement) are incorporated into every third training session on each robot module. These evaluations monitor participant performance and are used to give feedback and encouragement	At the initial therapy session a brief assessment of the participant’s upper limb is performed and up to 4 upper limb rehabilitation goals of importance to the participant are agreed. The activities to practise to achieve these goals are subsequently determined. Activities are divided into two types: whole-task or a part-task. Whole-task activity practice consists of practising all of the components of the task in sequence. Part-task activity practice consists of practising a specific part of a task. Part-task practice is appropriate if a participant has difficulty with a specific part of a task as it will enable them to focus on this particular aspect while working towards completing the task as a whole. The order to practise the activities and the time spent on each activity is at the discretion of the therapist and participant according to the participant’s rehabilitation priorities. Where appropriate, participants undertake a brief warm up consisting of gentle stretching of the upper limb, prior to practising the chosen activities. At the second and subsequent therapy sessions, following a brief warm up (if necessary), practice of the selected activities continues, with the order to practise and time to spend on each activity being at local discretion. At therapy sessions 12 (end of week 4) and 24 (end of week 8), progress towards goals is reviewed. If the participant has achieved a goal, a new goal is set and a new activity to practise selected. If the participant finds a goal or activity too challenging or they are experiencing other problems, an alternative is chosen. At the final therapy session (36, end of week 12), practice of activities continues but part of the session is dedicated to ‘summing up’ with feedback to the participant about progress over the programme and advice about maintaining upper limb function in the longer term
5. Who provided?: *‘for each category of intervention provider (for example, psychologist, nursing assistant) describe their expertise, background and any specific training given’*	A senior therapist (physiotherapist or occupational therapist) assesses each participant at their initial session on each robot module to ensure correct positioning and familiarisation with the robot. Therapy assistants (NHS band 3 or above) then deliver the robot-assisted training programme with senior supervision and support. A senior therapist reviews each participant at their last robot-assisted training session and provides feedback. All staff involved in the study received study-specific training. The senior therapists and therapy assistants delivering the robot-assisted training programme receive specific training in this aspect	A senior therapist (physiotherapist or occupational therapist) assesses each participant at their initial therapy session and they jointly select up to 4 upper limb rehabilitation goals and activities to practise. Therapy assistants (NHS band 3 or above) then deliver the enhanced upper limb therapy programme with senior supervision and support. A senior therapist reviews the participant every 4 weeks to plan/adjust the programme according to progress. A senior therapist reviews each participant at their last enhanced upper limb therapy session and provides feedback. All staff involved in the study received study-specific training The senior therapists and therapy assistants delivering enhanced upper limb therapy receive specific training in this aspect
6. How?: *‘describe the modes of delivery (such as face to face or by some other mechanism, such as Internet or telephone) of the intervention and whether it was provided individually or in a group’*	1:1 face-to-face delivery	1:1 face-to-face delivery
7. Where?: *‘describe the type of location(s) where the intervention occurred’*	NHS hospital facilities: dedicated therapy room	NHS hospital facilities: therapy gym or dedicated therapy room
8. When and how much?**:** *‘describe the number of times the intervention was delivered and over what period of time including the number of sessions, their schedule, duration, intensity or dose’*	The robot-assisted training programme is provided for up to 45 min per day, 3 days per week for 12 weeks (a total of 36 therapy sessions), in addition to usual NHS care. One hour is allowed for each therapy session to facilitate preparation and set up	The enhanced upper limb therapy programme is provided for up to 45 min per day, 3 days per week for 12 weeks (a total of 36 therapy sessions), in addition to usual NHS care. One hour is allowed for each therapy session to facilitate preparation and set up
9. Tailoring: *‘If intervention was planned to be personalised or adapted, then describe what, why, when and how’*	The use of the robot modules and order of blocks are standardised for all participants following the robot-assisted training programme. The only exception is in block 3 whereby if a participant is unable to use the hand module, the shoulder-elbow module is used on the ‘assist-as-needed’ random protocol (as described in section 4 above). The InMotion robotic gym system ‘assists-as-needed’ based on the specific performance of each patient. The system is designed to adjust the parameters (i.e. robot power and initiation of movement) as necessary during the therapy	The enhanced upper limb therapy programme is based on patient-centred goals. Up to 4 goals can be chosen. A senior therapist assesses/reviews each participant at baseline, 4, 8 and 12 weeks and plans/adjusts the programme according to progress. The therapists are also able to tailor the specifics of each activity practised to the ability of the participant, taking into consideration a range of upper limb parameters (i.e. sensation and proprioception, range of motion, strengths and coordination), other functions (including sitting balance, visuo-spatial awareness, vision), as well as the person’s cognitive and emotional status, communication skills and level of motivation
10. Modifications: *‘If intervention was modified during the course of the study, describe the changes what, why, when and how’*	There have been no modifications to the interventions to date	
11. How well (planned)?: *‘If intervention adherence or fidelity was assessed, describe how and by whom, and if any strategies were used to maintain or improve fidelity, describe them’*	Therapists/therapy assistants complete a robot-assisted training checklist to document attendance at sessions. The robot software records data on the robot protocol used, duration of the sessions and the number of repetitions of upper limb movements undertaken. Data from training sessions are periodically reviewed to monitor intervention adherence and feedback is provided to therapy staff delivering the intervention	Therapists/therapy assistants record session attendance, session duration, the type and number of goals, the type of activity practice (whole-task or part-task) and number of repetitions of each task at each session. Goal attainment is documented at each review session. Data from therapy sessions are periodically reviewed to monitor intervention adherence and feedback is provided to therapy staff delivering the intervention
12. How well (actual)?: *‘If intervention adherence or fidelity was assessed, describe the extent to which the intervention was delivered as planned’*	Unable to address until completion of study	

#### Enhanced upper limb therapy programme

The enhanced upper limb therapy programme aims to match the frequency and duration of the robot-assisted training programme sessions. It has been developed from the upper limb therapy programmes used in the Botulinum Toxin for the Upper Limb after Stroke (BoTULS) trial [27–29] and the Repetitive Arm Functional Tasks after Stroke (RAFTAS) project [30]. Using the principles of person-centred goal-setting and repetitive functional task practice, it aims to drive neuroplasticity and motor recovery after stroke.

Participants receive enhanced upper limb therapy at the hub sites for up to 45 min per day, 3 days per week for 12 weeks, in addition to usual care. A detailed description of the enhanced upper limb therapy programme using the TIDieR Checklist [26] is provided in Table 1.

#### Usual care

Defining usual care is a challenge for any stroke rehabilitation trial. One of the current NICE quality standards is that ‘patients with stroke should be offered a minimum of 45 min of each appropriate therapy that is required, for a minimum of 5 days a week, at a level that enables the patient to meet their rehabilitation goals for as long as they are continuing to benefit from therapy and as long as they are able to tolerate it’ [31]. For most stroke services this is aspirational and the majority of patients do not receive this intensity particularly after discharge from hospital or early supported discharge services [32]. Patients with chronic stroke are unlikely to receive ongoing rehabilitation in the longer term. Most services do not regularly review patients to address unmet rehabilitation needs beyond 1 year.

Usual care is delivered at hub and spoke sites.

Participants in all three randomisation groups receive a study ‘arm rehabilitation therapy log’ where they are asked to record any ‘usual’ upper limb rehabilitation that they receive during the course of the study. Periodic text message reminders are sent to remind participants about completion of the rehabilitation logs. In addition, participants in all three randomisation groups receive regular study newsletters.

### Outcome assessments

Outcomes are assessed at 3 months (±7 days) and 6 months (±7 days) following randomisation.

Assessments are undertaken in two stages:

Stage 1 is a self-completion postal questionnaire consisting of the Stroke Impact Scale (SIS) [33] (3 and 6 months) and the adapted Client Services Receipt Inventory resource utilisation questions (6 months only) [20–22].

Stage 2 is a face-to-face assessment with a researcher blinded to randomisation group. The following data are collected: Barthel ADL Index [16, 17], EQ-5D-5L [18], ARAT [11], Fugl-Meyer Test (motor and sensory arm sections [15]), and adverse events. At the end of the 6-month stage-2 assessment, participants are given a further self-completion questionnaire and are asked to return this by post. This questionnaire contains time and travel resource use questions [34, 35].

### Blinding

Due to the nature of the interventions, it is not possible to blind participants or treating therapists to treatment allocation. It is intended that stage-2 outcome assessments are conducted by a researcher blinded to treatment allocation. After each outcome assessment the researcher is asked to record whether they have unintentionally become aware of treatment allocation due to conversation with the participant. Success of outcome assessment blinding will be reported.

### Study withdrawal

No specific withdrawal criteria have been pre-set. Participants may withdraw from the study at any time for any reason. Data collected prior to withdrawal will be used in the study analysis unless consent for this is specifically withdrawn. Should a decision to withdraw from the study be made, a reason for withdrawal is sought but participants can chose to withdraw without providing an explanation.

Investigators, GPs, stroke physicians and therapists may also withdraw participants from the study at any time if they feel it is no longer in their interest to continue; for example, because of intercurrent illness or adverse events.

### Safety evaluation

The safety of robot-assisted training, enhanced upper limb therapy and usual care is being evaluated by examining the occurrence of all adverse events and serious adverse events in accordance with National Research Ethics Committee (NRES) guidance for non-CTIMP trials [36].

### Statistical analysis

#### Primary analysis

The primary outcome is arm function measured by the ARAT [11] at 3 months. It has been suggested that the minimal clinically important difference for the ARAT is 10% of its range (6 points) [37] but a smaller treatment effect may be clinically beneficial in those with severe initial upper limb functional limitation who are likely to improve less than those with more moderate limitation. There will be a stepped approach to define ‘successful outcome’: baseline ARAT 0–7 must improve by 3 or more points; baseline ARAT 8–13 improve by 4 or more points; baseline ARAT 14–19 improve by 5 or more points; baseline ARAT 20–39 improve by 6 or more points. Analyses will be by intention-to-treat. Logistic regression will be used to compare the primary outcome (success) between the three randomisation groups at 3 and 6 months, adjusting for any imbalance in key covariates. The use of multilevel logistic models will be explored. It may be possible to fit three-level models (hubs, spokes and participants), but since there are only four centres with a hub, and a small number of stroke services accessing an InMotion robotic gym system at each hub, it may be necessary to fit a two-level model (stroke services and participants).

#### Secondary analyses

The secondary outcomes will be compared between the three groups at 3 and 6 months using multilevel linear regression adjusting for baseline values and key covariates.

We will consider any difference in attrition rates, and any nonrandomness of the attrition, when comparing outcomes between the three groups. The pattern of missing observations because of loss to follow-up will be examined to determine both the extent of missingness, and whether it is missing at random or is informative. If data are missing to a sufficient extent, the use of appropriate multiple imputation techniques will be considered. Although mortality is possible within the 6-month follow-up period, it is thought to be sufficiently uncommon that methods for joint modelling of survival and longitudinal data will not be necessary.

Further descriptive analyses will explore the relationship between the severity of baseline upper limb function and time since stroke upon the effectiveness of the intervention. There is not sufficient power to perform any formal subgroup analyses. The time pattern of upper limb recovery will be explored by extending the earlier multilevel models to include a further within-patient level (ARAT scores collected at baseline, 3 and 6 months). However, this will depend on the relationship being approximately linear.

#### Sample size

The sample size is 720 participants (240 participants per group). Responses from 216 participants in each randomisation group will provide 80% power (significance level of 1.67% because of multiple comparisons) to detect a 15% difference in ‘successful outcome’ between each of the three pairs of treatments (robot-assisted training, enhanced upper limb therapy, usual care). We have allowed for 10% attrition and inflated the sample size to 720 participants.

### Economic analysis

The economic analysis will include a detailed micro-costing analysis, economic evaluation and a longer-term economic model. This will be based upon both a ‘within trial’ analysis and a modelling exercise to explore costs and effects over the longer term. Analyses will be carried out from the perspective of the NHS and personal and social services, but we will also take a societal perspective by including costs borne by the participants and their informal carers. All relevant costs associated with providing the interventions will be measured, this will include the cost of using the InMotion robotic gym system, costed on a per patient basis. All costs will be derived using routine data sources [38] and study-specific estimates. Where appropriate, discounting will be applied to costs and outcomes [39]. Costs in the follow-up period will also be taken into account; this includes secondary care resource, e.g. inpatient stays and outpatient visits; primary care resource use, e.g. general practice, therapy visits and prescription costs. These data will be collected using a health service utilisation questionnaire (adaption of the Client Services Receipt Inventory [20–22]) administered at baseline and 6 months post randomisation. Patient costs will also be collected via a time-and-travel questionnaire based upon one successfully used in a number of previous NIHR HTA-funded trials [34, 35]. This will include questions relating to travel time, time away from employment (if appropriate) and time spent providing care. The within-trial analysis will also compare changes in health-related quality of life, based on responses to the EQ-5D-5L at baseline, 3 and 6 months post randomisation and scored using population tariffs [40]. These data will be combined with study participants’ mortality to estimate quality-adjusted life years (QALYs). This measure provides a profile of quality of life over time. The results of the analyses will be presented as point estimates of mean incremental costs and QALYs. Techniques, such as bootstrapping, will be used alongside deterministic sensitivity analyses to address uncertainty [41]. In addition, a within trial cost-utility analysis will be performed where both costs and QALY data will be combined into an incremental cost per QALY. The cost-utility analysis will include deterministic and stochastic sensitivity analysis, presented as point estimates and cost-effectiveness acceptability curves (CEACs).

An economic model will also be developed to assess the cost and health consequences measured in terms of QALYs of stroke recovery beyond the 6-month timeframe of the trial. The data from the trial will be the main source of data for this model but further data with which to model outcomes beyond a 6-month follow-up will be systematically derived from the academic literature and other existing data sources following guidance for best practice [42]. These data will include information on factors, such as the incidence of hospitalisation and the need for residential/nursing home care, beyond the trial follow-up period. Sensitivity analysis will be applied to the model using probabilistic and deterministic sensitivity analyses to address parameter and other forms of uncertainty. The data on both costs and QALYs for both trial- and model-based analyses will be reported separately.

### Parallel process evaluation

Alongside the RCT, a two-stage process evaluation is being conducted to understand both (1) participants‘ and health service professionals’ experiences of robot-assisted training; enhanced upper limb therapy and usual care and (2) factors affecting the implementation of the trial within and across study sites. The process evaluation will capture data concerning feasibility and accumulating experience of the therapies being provided. In stage 1 data collection is by semi-structured interview using a pre-developed and piloted interview schedule. Data collection in stage 2 is primarily by interview; however, analysis also draws upon trial data including baseline, therapy and outcome (3 and 6 month) assessments. Interviews are primarily being conducted face to face; however, due to the geographical spread of the study sites, some follow-up interviews are being conducted by telephone for efficiency (these are particularly appropriate for health service professionals). Data collection and analysis relating to study of implementation factors will be informed by Normalization Process Theory (NPT) [43].

#### Participant study group

In stage 1 a subset of approximately 25–30 study participants will be recruited across study sites, to achieve a maximum variation sample, ensuring representation of participants differing in terms of key factors such as randomisation group, clinical severity and time from stroke. Participants in the robot-assisted training and enhanced upper limb therapy programme groups are interviewed on two occasions: (1) soon after therapy commences and (2) towards the end of the 12-week therapy period, to determine how perceptions of acceptability of therapy may change over time.

In stage 2 approximately 25 participants will be recruited, again with the aim of achieving maximum variation in the sample. Participants in the treatment groups are interviewed twice. However, in this stage, time points are (1) towards the end of their 12-week therapy and (2) around their 6-month follow-up assessments, to provide insight into their experience of trial participation, and the impact of the therapy they received, post treatment. The baseline, therapy and outcome assessment data are reviewed descriptively, for the participants who have been interviewed as part of stage 2. This will allow comparison of trial participants’ assessment data with their subjective experiences of participating in the trial, to inform later interpretation of the results of the trial.

Participants to be invited for interview are identified from the study database (containing data held by unique study number only) by the researcher conducting the interviews. The researcher advises the local study centre coordinators/administrators of the selected participant numbers and the local study coordinator/administrator/LCRN staff mails an invitation letter, an Information Sheet and a self-completion Contact Details Form for the participant to return directly to the researcher if they are interested in taking part in the interview(s).

The researcher telephones the responding participants, describes the purpose of the interview(s) and agrees a mutually convenient time for a first interview to take place. Prior to any potential second interview, participants are re-contacted by the researcher to check that they are still willing to take part in the second interview. Consent to be interviewed is obtained in writing prior to commencement of each interview.

#### Health service professional study group

A sample of approximately 20 health service professionals is being recruited across study sites and study groups. Interviews take place in stage 1 and stage 2 of the process evaluation. The aim is to interview a range of health service professionals, e.g. senior therapists, therapy assistants, study administrators, principal investigators and NIHR LCRN staff to gain insight into different aspects of the trial including implementation of the robot-assisted training, enhanced upper limb therapy and usual care practices, and implementation of the trial itself, including the recruitment and follow-up processes.

Staff to be invited for interview are identified by the local study centre coordinator and/or local study investigators. Each selected member of staff receives a letter of invitation and an Information Sheet. Following issue of the invitation letter and Information Sheet, the researcher conducting the interviews contacts the selected staff to go over the purpose of the interviews and ascertain willingness to take part. A mutually convenient time and place for the interview(s) is agreed. Consent to be interviewed is obtained in writing prior to commencement of each interview.

#### Interview data analysis

Interviews are audio-taped with the respondents’ consent, and transcribed. Data will be mostly analysed using the constant comparative method of qualitative analysis [44] facilitated by analysis software (QSR NVivo). For a subset of the process evaluation data – that specifically focussed on questions concerning implementation – a theory-based approach to analysis will be undertaken [43]. All data analysis will include a proportion of data to be analysed collectively in ‘data clinics’ where the research team share and exchange interpretations of key themes emerging from the data. A larger proportion of data, however, will be independently thematically coded and compared between two researchers to ensure consistency in the interpretation of data within a broader thematic framework developed as data collection progresses.

### Confidentiality

Personal data are regarded as strictly confidential. Original paper Case Record Forms containing study data are stored in the investigator site file at each research site. All study files are securely stored and access restricted to staff involved in the study. Research staff at sites enter data from paper forms onto a secure web-based electronic database run and maintained by Newcastle University. Data are entered using participant-unique study numbers only. Access to this database is password-protected and limited to staff at research sites or Newcastle University who are involved in the study.

The InMotion robotic gym computers store data from each participant session. Data are stored by unique study number only. Periodically, these data are copied from the robot computer system into an electronic database maintained by Newcastle University.

The study complies with the Data Protection Act 1998, and Caldicott Guardian approval for use of patient identifiable data.

### Trial monitoring, quality control and quality assurance

The chief investigator has overall responsibility for study conduct. The principal investigators are responsible for the day-to-day study conduct at their individual sites.

The trial is managed by a coordinating centre based at Newcastle University which provides day-to-day support for the sites and provides training through investigator meetings, site initiation visits and routine monitoring visits. A Trial Management Group (TMG) has been convened and the TMG meets regularly during the study.

Quality control is maintained through adherence to Newcastle Biomedicine Clinical Research Platform SOPs, the study protocol and research governance regulations. General monitoring of study conduct and data collected is being performed by a combination of central review and site-monitoring visits. The main areas of focus include consent, serious adverse events and essential documents in study files. All monitoring findings are reported and followed up with the appropriate persons in a timely manner.

A Trial Steering Committee (TSC) has been convened. This comprises an independent chair, three other independent members, a patient and/or a carer representative and the chief investigator. The TSC has agreed a charter of operation and meet at least annually.

An independent Data Monitoring and Ethics Committee (DMEC) has been convened to undertake independent review. This comprises five independent members including expert health care professionals and a statistician. Only the DMEC has access to unblinded outcome data before the trial ends. The DMEC has agreed a charter of operation and meets at least annually.

### Dissemination of results

The data are the property of the chief investigator and co-investigator(s). Publication will be the responsibility of the chief investigator.

The study will be presented at national and international conferences, and reported in peer-reviewed journals and a NIHR HTA monograph. Reports will be written for the study sponsor and regulatory bodies. A summary of the results will be sent to study participants and be available on the study website: https://research.ncl.ac.uk/ratuls/.

Anonymised data will be provided to research databases as requested (e.g. the Cochrane Collaboration, the Virtual International Stroke Trials Archive (VISTA)) to enable future meta-analyses. Anonymised robot kinematic and kinetic data will be provided to co-investigators for exploratory analyses.

## Discussion

Robot-assisted training is a promising treatment for improving the upper limb function of patients with moderate to severe upper limb impairment post stroke, but further high-quality research is needed before this technology should be integrated into clinical practice [8].

RATULS is a large, multicentre RCT to determine whether robot-assisted training with the InMotion robotic gym system (MIT-Manus commercial version) improves upper limb function post stroke when compared to an upper limb therapy programme of the same frequency and duration of sessions and usual NHS care. The results from the trial will inform clinicians and commissioners of health care about the clinical effectiveness and cost-effectiveness of robot-assisted training.

### Trial status

RATULS commenced recruitment in April 2014. Four NHS study centres (Glasgow, North Tyneside, Northwick Park, and Romford) are participating. The RATULS trial has recruited 468 patients at the time of submission of this manuscript. Protocol version 3, dated 2 August 2016, was used to prepare the manuscript.

## Additional file

Additional file 1: SPIRIT Checklist for RATULS protocol paper. (DOC 120 kb)

## Abbreviations

ADL: Activities of daily living
ARAT: Action Research Arm Test
CEA: Cost-effectiveness analysis
CEAC: Cost-effectiveness acceptability curve
CSRI: Client Services Receipt Inventory
GP: General practitioner
LCRN: Local Clinical Research Network
NHS: National Health Service
NICE: National Institutes for Health and Care Excellence
NIHR: National Institutes for Health Research
NIHR HTA: National Institutes for Health Research Health Technology Assessment
NIHSS: National Institutes of Health Stroke Scale
NRES: National Research Ethics Service
QALY: Quality-adjusted life year
RCT: Randomised controlled trial
SIS: Stroke Impact Scale
UK: United Kingdom
